# Waist circumference increases risk of coronary heart disease: Evidence from a Mendelian randomization study

**DOI:** 10.1002/mgg3.1186

**Published:** 2020-02-24

**Authors:** Qinchang Chen, Lingling Li, Junzhe Yi, Kai Huang, Runnan Shen, Ridong Wu, Chen Yao

**Affiliations:** ^1^ Department of Vascular Surgery The First Affiliated Hospital Sun Yat‐sen University Guangzhou China; ^2^ Department of Vascular Surgery Sun Yat‐sen Memorial Hospital Sun Yat‐sen University Guangzhou China; ^3^ Zhongshan School of Medicine Sun Yat‐sen University Guangzhou China

**Keywords:** coronary heart disease, Mendelian randomization, waist circumference

## Abstract

**Background:**

This study investigated whether expanding waist circumference (WC) is causally associated with an elevated risk of coronary heart disease (CHD), using a two‐sample Mendelian randomization (MR) study through integrating summarized data from genome‐wide association study.

**Methods:**

The data included in this analysis were mainly from the Genetic Investigation of ANthropometric Traits (GIANT), Consortium and Coronary Artery Disease Genome wide Replication, and Meta‐analysis plus the Coronary Artery Disease (C4D) Genetics (CARDIoGRAMplusC4D) Consortium. Three statistical approaches, inverse‐variance weighted (IVW), weighted median, and MR‐Egger regression method were conducted to assess the casual relationship. The exposure was WC, measured by 46 single‐nucleotide polymorphisms from GIANT and the outcome was the risk of CHD. Then, we used the genetic data from Neale Lab and TAG to infer whether WC causally affected the established risk factors of CHD.

**Results:**

The IVW method presented that genetically predicted WC was positively casually associated with CHD (odds ratio [OR]: 1.57, 95% CI = 1.33–1.84; *p* = 4.81e‐08), which was consistent with the result of weighted median and MR‐Egger regression. MR‐Egger regression indicated that there was no directional horizontal pleiotropy to violate the MR assumption. Additionally, expanded WC was also associated with higher risk of hypertension and diabetes, higher cholesterol, more smoking intensity, and decreased frequency of physical activity.

**Conclusion:**

Our analysis provided strong evidence to indicate a causal relationship between WC and increased risk of CHD.

## Graphical abstract

Our results indicated that 12.5 cm increased waist circumference (WC) predicted more than 1.5‐folds increased risk of coronary heart disease (CHD). We have identified that lager WC was statistically associated with hypertension, diabetes, smoking, high cholesterol, and decreased physical activity, which were established risk factors of CHD.

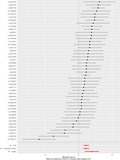

## 1 INTRODUCTION

Coronary heart disease (CHD), also called coronary artery disease, refers to the cardiovascular disease with a narrowing or blockage of the coronary arteries, which is the major cause of mortality and disability worldwide (Abraham et al., 2016; Mahmood, Levy, Vasan, & Wang, 2014; Mozaffarian et al., 2015). Globally, CHD led to more than 17 million deaths in 2016 and most of them were in developed countries (Eriksson et al., 2018; Fung, Isanaka, Hu, & Willett, 2018). Moreover, the mortality is also increasing in developing countries. Most cardiovascular diseases are asymptomatic, indicating the importance of early detection and risk factors avoidance. It is believed that addressing behavioral risk factors helps to the prevention of CHD, such as tobacco use, physical inactivity, harmful use of alcohol, and so on (Hackshaw, Morris, Boniface, Tang, & Milenković, 2018; Logue et al., 2011; De Schutter, Lavie, & Milani, 2014). Smoking is one of the most important risk factors for CHD. Though smoking few cigarettes is generally considered as safe, recent research found that there was no safe zone of cigarette smoking for cardiovascular disease (Hackshaw et al., 2018). However, smoking is not the only risk factor and CHD in nonsmokers is also the severe problem. With clinical development of cardiovascular researches, CHD affected by multiple risk factors is gradually been recognized. As a result, more researches are needed to explore more potential risk factors, which is important for harm reduction in CHD.

Obesity has been shown strongly associated with the elevated risk of CHD in several clinical studies (Coutinho et al., 2011; Flint et al., 2010; Li et al., 2006; Logue et al., 2011; De Schutter et al., 2014). Along with the deepened study gradually, central obesity but not BMI is more likely directly associated with mortality of CHD (Coutinho et al., 2011). Waist circumference (WC) is the important indicator of central obesity. After adjusting the other risk factors for CHD, WC remains the strong predictor of CHD risk (Lofgren et al., 2004). However, due to the methodological characteristic of conventional observational studies, reverse causation and residual confounding are inevitable (Boyko 2013). Therefore, understanding of the effect of WC on CHD risk is limited and the casual relationship remains unclear. Randomized controlled trial (RCT) is the gold standard to perform inference of causal association. However, for the characteristic of chronic disease, there might be long period between the exposure and the emergence of outcome. If RCT is selected to investigate this issue, large time and economic cost will be spent and it is unethical for the population with huge WC but without any medication. Recently, genetic variants can be selected as the instrumental variable of exposure in Mendelian randomization (MR) study, which has robuster ability of interference than conventional study (Davey Smith & Ebrahim 2003).

MR study is an epidemiological research by using the genetic variants as instrumental variable to investigate the casual relationship between exposures and diseases (Burgess, Butterworth, & Thompson, 2013). Compared with the conventional study, MR study is able to overcome the epidemiological limitations, such as reverse causation, measurement error and so on (Lawlor 2016). Recently, with the development of genome‐wide association studies (GWAS), MR study can draw on the new information from GWAS to investigate the genetic variants and exposures (Benn & Nordestgaard 2018).To expand the statistical power in MR study, investigators explore the association of instrument‐exposure and instrument‐outcome association results, respectively, in different samples to infer the casual effect, also called two‐sample MR study (Hartwig, Davies, Hemani, & Davey Smith, 2016). In view of the unique advantages of MR in clinical research, MR has been applied in many diseases, such as CHD, lung cancer, hypertension, and so on (Interleukin‐6 Receptor Mendelian Randomisation Analysis (IL6R MR) Consortium, 2012; C Reactive Protein Coronary Heart Disease Genetics Collaboration, 2011; Vimaleswaran et al., 2014; Gao et al., 2016). Nevertheless, no researches have been reported as the casual association between WC and CHD by the method of MR.

In this study, we aimed to perform a two‐sample MR study to assess the causal association between WC and risk of CHD.

## 2 METHODS

### 2.1 Genetic variants associated with WC

Data for the single‐nucleotide polymorphisms (SNPs) associated with WC were the GWAS summary statistics datasets from the Genetic Investigation of ANthropometric Traits (GIANT) Consortium (https://www.broadinstitute.org/collaboration/giant/index) (Shungin et al., 2015). This consortium contained 224,459 participants, as well as 2,566,630 SNPs. Details of consortium were presented in Table 1. In order to avoid the bias caused by strong linkage disequilibrium among SNPs, we identified the SNPs associated with WC at a GWAS threshold of statistical significance (*p* < 5 × 10^−8^; linkage disequilibrium *r* ^2^ < 0.1). Finally, there were forty‐six SNPs identified and included in this MR analysis (rs10132280, rs10786712, rs10840100, rs10938397, rs11039290, rs11165623, rs11676272, rs13404250, rs1412235, rs1421085, rs1519480, rs16996700, rs17066856, rs17381664, rs17724992, rs1928295, rs2033529, rs2278076, rs2287019, rs2307111, rs2325036, rs2357760, rs2531992, rs2867110, rs3127553, rs3800229, rs4234589, rs4788099, rs4883723, rs4985155, rs543874, rs6440003, rs6545714, rs6567160, rs6864049, rs7138803, rs7144011, rs7239883, rs749671, rs7531118, rs7550711, rs7649970, rs7903146, rs806794, rs929641, and rs943005). Forty‐six included SNPs explained 0.093﹪ of the variation in the WC among the participants. In this analysis, the *F* value was larger than 10, which meant that the MR analysis was able to avoid bias from week instrument (Burgress et al., 2011). SNPs were selected as the strong instrument variables in the MR analysis.

**Table 1:** Details of studies and datasets used for analyses

Exposure/outcome	Number of cases	Number of controls	Sample of size	Web source	First author	Consortium	Year	Population
Waist circumference	N/A	N/A	224,459	https://www.ncbi.nlm.nih.gov/pubmed/25673412	Shungin D	GIANT	2015	Mixed
Coronary heart disease	60,801	123,504	184,305	https://www.ncbi.nlm.nih.gov/pubmed/26343387	Nikpay	C4D	2015	Mixed
Hypertension	87,690	249,469	337,159	http://www.nealelab.is/blog/2017/9/11/details-and-considerations-of-the-uk-biobank-gwas	Neale	Neale Lab	2017	European
Diabetes	16,183	320,290	336,473	Data from http://www.nealelab.is/blog/2017/9/11/details-and-considerations-of-the-uk-biobank-gwas	Neale	Neale Lab	2017	European
Cigarettes smoked per day	N/A	N/A	68,028	https://www.ncbi.nlm.nih.gov/pubmed/20418890	Furberg	TAG	2010	European
High cholesterol	41,296	295,863	337,159	Data from http://www.nealelab.is/blog/2017/9/11/details-and-considerations-of-the-uk-biobank-gwas	Neale	Neale Lab	2017	European
Number of days/weeks Moderate physical activity	N/A	N/A	321,309	Data from http://www.nealelab.is/blog/2017/9/11/details-and-considerations-of-the-uk-biobank-gwas	Neale	Neale Lab	2017	European

Abbreviations: N/A, not available; GIANT, Genetic Investigation of ANthropometric Traits.

### 2.2 GWAS summary data on CHD

We obtained the GWAS summary data on CHD from Coronary Artery Disease Genome‐wide Replication and Meta‐analysis plus the Coronary Artery Disease (C4D) Genetics (CARDIoGRAMplusC4D) Consortium (http://www.cardiogramplusc4d.org/), which contained 60,801 cases and 123,504 controls (Locke et al., 2015). The corresponding information of 46 SNPs included in this study could be found in this database. Therefore, all the SNPs were included in the final MR analysis.

### 2.3 Effect estimation

To assess the casual relationship between WC and CHD, we conducted three statistical approaches, inverse‐variance weighted (IVW), weighted median, and MR‐Egger regression method. The premise is that all SNPs are valid tool variables. Under the premise of IVW applications that all SNPs are valid genetic variables, IVW is performed to infer the casual relationship through the meta‐analysis of the Wald ratio for the included SNPs (Burgess et al., 2013). The result of weighted median is median when effect estimation of individual SNP was ranked as the order of weight value (Bowden, Davey Smith, Haycock, & Burgess, 2016). When at least 50% of SNPs are valid variables, the weighted median method can provide a consistent estimate of the final effect. When the intercept term of the regression is zero, or the intercept term is not statistically significant, the slope of the MR‐Egger regression represents an estimate of the causal effect of the exposure on the outcome (Bowden et al., 2017). Characteristics of the SNPs associated with WC and with CHD were shown in Table 2.

**Table 2:** Characteristics of the single‐nucleotide polymorphisms associated with waist circumference and with coronary heart diseases

SNP	EA	EAF	Associations with WC			Associations with CHD		
			Beta	SE	*p*	Beta	SE	*p*
rs10132280	A	0.282	−0.022	0.004	1.10e‐09	0.012	0.011	.254
rs10786712	C	0.598	−0.018	0.003	4.10e‐08	0.043	0.009	5.08e‐06
rs10840100	G	0.608	0.019	0.003	1.20e‐08	0.015	0.010	.125
rs10938397	A	0.584	−0.032	0.003	5.60e‐22	−0.031	0.009	.001
rs11039290	A	0.308	−0.022	0.004	1.50e‐09	−0.004	0.010	.724
rs11165623	A	0.464	0.021	0.003	3.90e‐10	0.008	0.009	.415
rs11676272	G	0.474	0.021	0.004	5.20e‐09	0.020	0.009	.028
rs13404250	C	0.679	0.021	0.004	1.60e‐09	0.026	0.010	.011
rs1412235	C	0.293	0.024	0.004	6.90e‐12	0.023	0.010	.025
rs1421085	C	0.380	0.072	0.003	1.30e‐102	0.030	0.010	.002
rs1519480	C	0.350	0.03	0.004	2.90e‐18	0.024	0.010	.016
rs16996700	T	0.741	0.022	0.004	4.90e‐10	0.004	0.011	.722
rs17066856	C	0.105	−0.035	0.006	1.90e‐10	−0.038	0.015	.015
rs17381664	C	0.349	0.022	0.004	4.00e‐10	0.016	0.010	.113
rs17724992	A	0.706	0.021	0.004	2.40e‐08	0.014	0.010	.174
rs1928295	C	0.437	−0.018	0.003	1.70e‐08	−0.007	0.010	.469
rs2033529	G	0.266	0.02	0.004	1.80e‐08	−0.006	0.010	.547
rs2278076	A	0.240	−0.024	0.004	2.50e‐09	−0.013	0.011	.220
rs2287019	C	0.817	0.033	0.004	4.70e‐14	0.039	0.013	.002
rs2307111	C	0.443	−0.025	0.003	1.10e‐13	−0.008	0.010	.429
rs2325036	A	0.587	0.021	0.003	1.10e‐10	0.001	0.009	.879
rs2357760	A	0.651	0.019	0.003	4.10e‐08	0.017	0.010	.088
rs2531992	A	0.215	−0.027	0.005	2.70e‐09	0.004	0.012	.768
rs2867110	C	0.167	−0.051	0.004	2.30e‐32	−0.040	0.012	.001
rs3127553	G	0.402	0.022	0.003	5.20e‐11	0.010	0.010	.281
rs3800229	T	0.653	0.023	0.004	5.60e‐11	0.004	0.010	.694
rs4234589	G	0.127	−0.032	0.005	9.60e‐11	−0.011	0.014	.412
rs4788099	G	0.346	0.034	0.003	2.00e‐24	0.016	0.010	.112
rs4883723	G	0.834	−0.028	0.005	1.30e‐09	−0.007	0.013	.580
rs4985155	A	0.640	0.019	0.003	3.00e‐08	−0.006	0.010	.500
rs543874	G	0.189	0.044	0.004	2.00e‐26	0.008	0.012	.517
rs6440003	G	0.579	−0.021	0.003	9.10e‐11	0.017	0.010	.072
rs6545714	G	0.386	0.022	0.003	5.40e‐11	0.010	0.010	.288
rs6567160	C	0.254	0.05	0.004	5.80e‐38	0.058	0.011	4.56e‐08
rs6864049	A	0.445	−0.019	0.003	2.40e‐08	−0.004	0.010	.671
rs7138803	G	0.628	−0.028	0.003	1.60e‐16	−0.008	0.010	.388
rs7144011	T	0.202	0.032	0.004	1.60e‐15	0.021	0.012	.076
rs7239883	G	0.388	0.019	0.003	5.90e‐09	0.002	0.010	.866
rs749671	A	0.361	−0.02	0.003	4.80e‐09	−0.010	0.010	.308
rs7531118	T	0.519	−0.027	0.003	5.40e‐15	−0.018	0.010	.069
rs7550711	T	0.028	0.057	0.010	3.10e‐09	−0.048	0.031	.114
rs7649970	T	0.125	0.027	0.005	1.90e‐08	−0.001	0.014	.969
rs7903146	T	0.276	−0.022	0.004	1.70e‐09	0.033	0.010	.001
rs806794	G	0.330	−0.022	0.004	1.50e‐09	−0.013	0.010	.185
rs929641	A	0.573	0.019	0.003	7.70e‐09	0.015	0.010	.122
rs943005	T	0.182	0.039	0.004	7.00e‐20	0.018	0.012	.130

Abbreviations: CHD, coronary heart disease; SNP, single‐nucleotide polymorphism; WC, waist circumference.

### 2.4 Sensitivity analysis

Leave‐one‐out method was selected to analyze the sensitivity of the results, which sequentially removed one of the SNPs and used the remaining SNPs as instrumental variables for MR analysis, and estimated the total effect by IVW method (Mikshowsky, Gianola, & Weigel, 2017). Though the stability of the effect estimate, the extent to the individual SNP affecting the causal relevance estimate can be determined.

### 2.5 Risk factors analysis

In order to demonstrate the potential mechanisms between WC and CHD, IVW methods were selected to study the possible casual relationship between WC and the common risk factors of CHD, such as hypertension, diabetes, high cholesterol, smoking, and physical activity. The genetic information on hypertension, diabetes, high cholesterol, and physical activity was obtained for Neale Lab Consortium. The association of WC and cigarettes smoked per day was based on the data from Tobacco and Genetics Consortium (TAG) (Tobacco, 2010).

## 3 RESULTS

### 3.1 Casual effect from WC to CHDs

**Figure 1:**
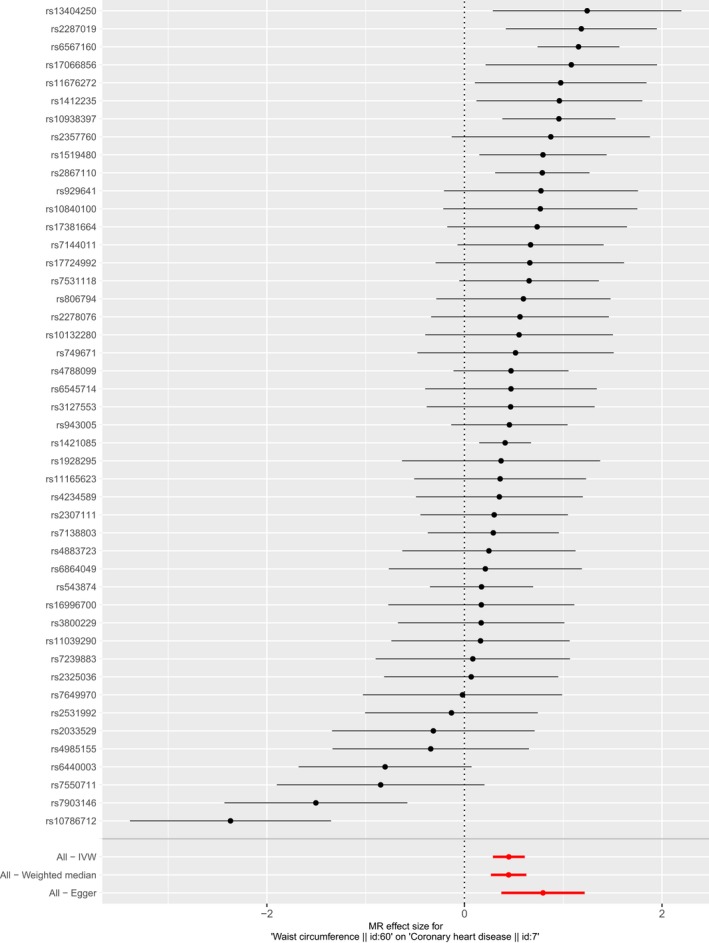
Forest plot of single‐nucleotide polymorphisms (SNPs) associated with waist circumference (WC) and their risk of CHD. Black points represent the log odds ratio (OR) for CHD per standard deviation (*SD*) increase in WC, which is produced by using each SNPs selected as separate instruments (rs10132280, rs10786712, rs10840100, rs10938397, rs11039290, rs11165623, rs11676272, rs13404250, rs1412235, rs1421085, rs1519480, rs16996700, rs17066856, rs17381664, rs17724992, rs1928295, rs2033529, rs2278076, rs2287019, rs2307111, rs2325036, rs2357760, rs2531992, rs2867110, rs3127553, rs3800229, rs4234589, rs4788099, rs4883723, rs4985155, rs543874, rs6440003, rs6545714, rs6567160, rs6864049, rs7138803, rs7144011, rs7239883, rs749671, rs7531118, rs7550711, rs7649970, rs7903146, rs806794, rs929641, and rs943005). Red points showing the combined causal estimate using all SNPs together in a single instrument, using two different methods (inverse‐variance weighted [IVW] approach, and MR‐Egger). Horizontal line segments denote 95% confidence intervals of the estimate. CHD, coronary heart disease; MR, Mendelian randomization

**Figure 2:**
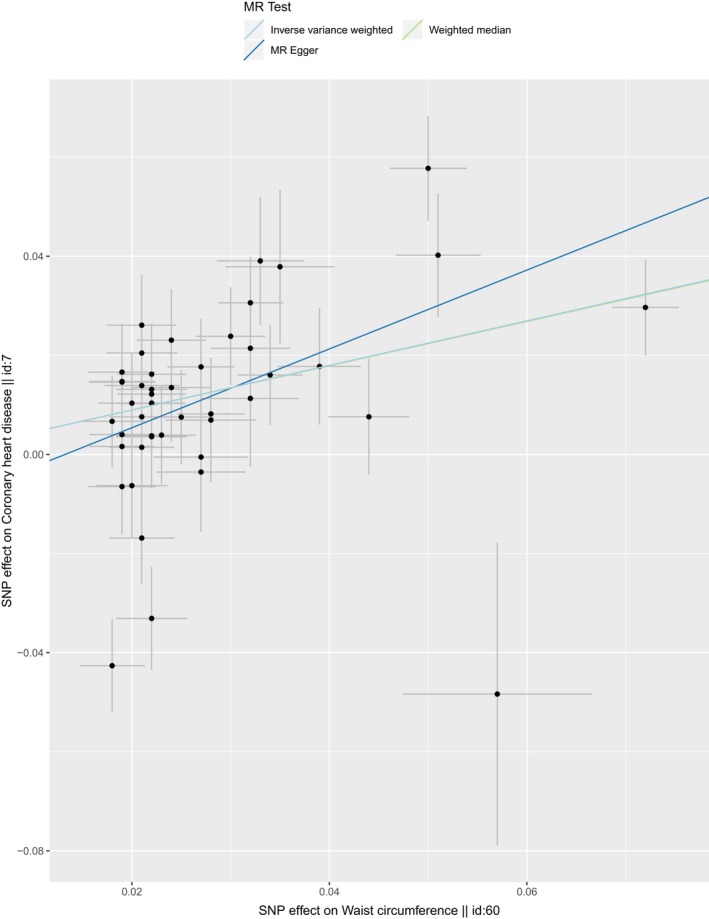
Scatter plot of SNPs associated with WC and the risk of CHD. The plot relating the effect sizes of the SNP‐LDL association (x‐axis, *SD* units) and the SNP‐CHD associations (y‐axis, log (OR)) with 95% confidence intervals. The regression slopes of the lines correspond to causal estimates using four of the Mendelian randomization (MR) methods. (IVW approach, MR‐Egger, and Weighted median). CHD, coronary heart disease; IVW, inverse‐variance weighted; SNP, single‐nucleotide polymorphism; WC, waist circumference

**Figure 3:**
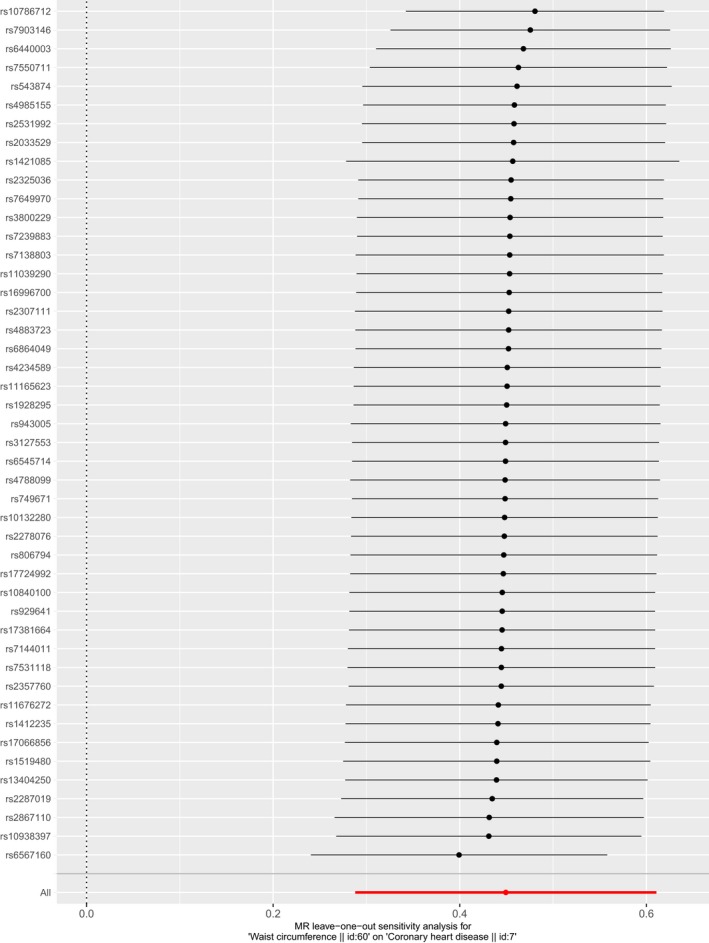
Leave‐one‐out of SNPs associated with WC and their risk of CHD. Each black point represents the IVW MR method applied to estimate the causal effect of LA on CHD excluding particular SNP (rs10132280, rs10786712, rs10840100, rs10938397, rs11039290, rs11165623, rs11676272, rs13404250, rs1412235, rs1421085, rs1519480, rs16996700, rs17066856, rs17381664, rs17724992, rs1928295, rs2033529, rs2278076, rs2287019, rs2307111, rs2325036, rs2357760, rs2531992, rs2867110, rs3127553, rs3800229, rs4234589, rs4788099, rs4883723, rs4985155, rs543874, rs6440003, rs6545714, rs6567160, rs6864049, rs7138803, rs7144011, rs7239883, rs749671, rs7531118, rs7550711, rs7649970, rs7903146, rs806794, rs929641, and rs943005, respectively) from the analysis. Each red point depicts the IVW estimate using all SNPs. No single SNP is strongly driving the overall effect of WC on CHD in this leave‐one‐out sensitivity analysis. CHD, coronary heart disease; IVW, inverse‐variance weighted; MR, Mendelian randomization; SNP, single‐nucleotide polymorphism; WC, waist circumference

The result of MR analysis indicated that the larger WC size was strongly associated with higher risk of CHD (Table 3). According to the result of IVW, one *SD* larger WC size (12.5cm) was associated with a 1.57‐fold risk of CHD (OR 1.20, 95% CI 1.33–1.84, *p* = 4.81e‐08; Q statistical power 103.1, *p* = 1.87e‐06). The harmful association was also observed in the result of weighted median and MR‐Egger method. The casual estimates were similar between the IVW method and weighted median while MR‐Egger provided a larger estimate. The individual causal effect of SNPs has been shown in Figure 1. Gene rs13404250, rs2287019, rs6567160, rs17066856, rs11676272, rs1412235, rs10938397, and rs1421085 showed significant effect on the association between WC and CHD. The regression slopes were illustrated in Figure 2. MR‐Egger regression results indicated that genetic pleiotropic effects did not bias the result (intercept = −0.013, *p* = .09), which was consistent with the hypothesis. In the leave‐one‐out analysis, there was no SNPs with strong effect on the overall estimates from WC to CHD. All the results in Figure 3 was consistent with the harmful effect.

**Table 3:** Mendelian randomization estimates of the associations between Waist circumference and risk of coronary heart diseases

Outcome	IVW method		MR‐Egger		Weight median method	
	OR (95％ CI)	*p*‐value	OR (95％ CI)	*p*‐value	OR (95％ CI)	*p*‐value
Coronary heart diseases	1.57 (1.33–1.84)	4.81e‐08*	2.22 (1.45–3.38)	5.96e‐04*	1.56 (1.30–1.88)	2.12e‐06*

Note

Test for horizontal pleiotropy: MR‐Egger intercept = −0.011, SE = 0.006, *p* = .090.

Abbreviations: CI, confidence interval; IVW, inverse‐variance weighted; MR, Mendelian randomization; OR, odds ratio.

* *p*‐valve < .05.

### 3.2 Casual effect from WC to risk factors

As shown in Table 4, one *SD* larger WC size (12.5cm) was statistically associated with a 1.09‐fold risk of hypertension, 1.04‐fold risk of diabetes, 1.03‐fold risk of high cholesterol, more smoking intensity [OR 6.94 (2.22, 21.68) Cigarettes smoked per day], and decreased frequency of physical activity [OR 0.89 (0.82,0.97) number of days/weeks Moderate physical activity].

**Table 4:** Causal effects from Waist circumference to common risk factors of coronary heart disease

Outcomes	Casual effect (95％CI)	*p*‐value
Hypertension	1.09 (1.06–1.12)	5.52e‐10
Diabetes	1.04 (1.01–1.07)	.003
Cigarettes smoked per day	6.94 (2.22–21.68)	8.62e‐04
High cholesterol	1.03 (1.00–1.04)	.002
Number of days/weeks Moderate physical activity	0.89 (0.82–0.97)	.008

* *p*‐value < .05.

### 3.3 Casual effect from CHD to WC

We have also conducted another mendelian randomization study to infer the casual relationship between CHD and WC. The result indicated that CHD was not causally associated with WC (OR 0.98, 95% CI = 0.94‐1.02, *p* = 0.33). The similar result was also observed in other statistical methods (Table 5).

**Table 5:** Mendelian randomization estimates of the associations between coronary heart diseases and waist circumference

Methods	OR (95% CI)	*p*‐value
IVW	0.98 (0.94–1.02)	.33
MR‐Egger	0.93 (0.83–1.03)	.16
Weighted median	0.98 (0.96–1.01)	.13

Abbreviations: CI, confidence interval; IVW, inverse‐variance weighted; MR, Mendelian randomization; OR, odds ratio.

* *p*‐valve < .05.

## 4 DISCUSSION

In this MR study, the major finding was that WC was casually associated with CHD. Our results indicated that 12.5cm increased WC predicted more than 1.5‐folds increased risk of CHD. Moreover, to further investigate the potential mechanism between WC and CHD, we have identified that lager WC was statistically associated with hypertension, diabetes, smoking, high cholesterol, and decreased physical activity, which were established risk factors of CHD (Table 5).

Obesity, the independent predictor of CHD, is an epidemic around the world and its prevalence increases dramatically among children and adults (Coutinho et al., 2011). Recently, there was increasing evidence to advocate that WC was associated with elevated morbidity and mortality of CHD patients. (Coutinho et al., 2011; Flint et al., 2010; Li et al., 2006; Lofgren et al., 2004). Alan and his colleagues demonstrated that WC strongly predicted future risk of CHD in two prospective cohort studies, the Health Professionals Follow‐up Study and the Nurses’ Health Study (Flint et al., 2010). A systematic review and collaborative analysis in subjects with 15,923 CHD have also consistently shown central obesity is directly associated with CHD (Coutinho et al., 2011). Our result was also in accord with the finding of Lofgren, whose researches supported that WC was a stronger predictor of CHD risk than BMI (Lofgren et al., 2004). For MR analysis, all of them have strong points and weaknesses for the prediction and bias control, for which we used three MR approaches to guarantee the casual inference between WC and CHD. In this analysis, all these approaches presented similarly statistically significant results to infer the association.

Our findings also demonstrated that WC was associated with established risk factors of CHD, such as hypertension, diabetes, high cholesterol, smoking intensity, and decreased physical activity, which might the potential mechanism underlying the casual association between WC and CHD. Hypertension, the major risk factor for CHD, is related to the activation of renin–angiotensin system (RAS) (Te Riet, Esch, Roks, Meiracker, & Danser, 2015). Apart from liver, adipose is another important source of angiotensinogen and expresses the receptor of angiotensin II, which lead to generate dysfunctional adipocytes (Te Riet et al., 2015; Bouchouirab, Fortin, Noll, Dubé, & Carpentier, 2018; LeMieux et al., 2016). Thus, the wretched cycle between RAS and dysfunctional adipocytes might be the possible mechanism for the obesity‐related hypertension. Insulin resistance was observed both in obesity and diabetes. The nonesterified fatty acids, secreted from adipose tissue in obesity, are considered as the core factors to cause insulin resistance (Bouchouirab et al., 2018). Additionally, body fat distribution is also the important factors to determine insulin resistance. Compared with subcutaneous fat, abdominal fat is more difficult to the antilipolytic function of insulin, for which, the abdominal fat plays an essential role in insulin resistance and diabetes (Bouchouirab et al., 2018; Roden et al., 1996). Dyslipidemia is another symptom of central obesity, such as high cholesterol, high hypertriglyceridemia, low high‐density lipoprotein, and so on, all of which might contribute to the cardiovascular events (Navab, Anantharamaiah, & Fogelman, 2005; Pausova 2006). Here, we also observed that larger WC was associated with the increase of smoking intensity. It is believed that smoking is effective to reduce body weight, whether smokers or nonsmokers (Potter, Pederson, Chan, Aubut, & Koval, 2004). The obese people, who are expected to lose weight, are more likely to start smoking. Concerns about weight and fear of weight gain are regarded as the important relapse reason for smoking, especially the young women (Potter et al., 2004). Obesity, especially the abdominal obesity, has been demonstrated with decreased physical activity and in turn, physically inactive lifestyle will continue to increase adiposity （Pietiläinen et al., 2008）. Physical inactivity has been identified as the risk factor for CHD for long and the effect of physical activity is emphasized in the prevention and treatment of CHD (Pietiläinen et al., 2008; Winzer, Woitek, & Linke, 2018). Though the casual association between WC and the risk factors of CHD has been revealed using MR analysis in our study further efforts are still needed to investigate the accurate degree of mediation from risk factors.

The study had some limitations. First, due to the limitations of the data we used, the association between WC and CHD was supposed as linear relation. Concerning that underweight is also the risk factor for CHD, the association might be U‐shaped and further researches are needed (Suastika et al., 2012). Second, the data used to infer the association between WC and risk factors of CHD were mainly from European consortiums. Thus, the generalizability of our findings has still to be proved. Third, we are not able to perform subgroup analysis on the interest covariates through the summary data, without individual patient data from the consortiums.

## 5 CONCLUSION

In summary, our analysis provided strong evidence to indicate a causal relationship between WC and increased risk of CHD. Further researches are needed to validate our finding and explore the potential mechanism.

## CONFLICT OF INTEREST

The authors declare that they have no competing interests.

## ETHICS APPROVAL AND CONSENT TO PARTICIPATE

Not applicable.

## Data Availability

These data were derived from the following resources available in the public domain: The GIANT Consortium (https://www.broadinstitute.org/collaboration/giant/index), Coronary Artery Disease Genome wide Replication and Meta‐analysis plus the Coronary Artery Disease (C4D) Genetics (CARDIoGRAMplusC4D) Consortium (http://www.cardiogramplusc4d.org/), Neale Lab Consortium (https://doi.org/10.1111/j.1572-0241.2000.02094.x), and Tobacco and Genetics consortium (TAG) (https://doi.org/10.1038/ng.571.).
